# Influence of exposing dental implants into the sinus cavity on survival and complications rate: a systematic review

**DOI:** 10.1186/s40729-019-0157-7

**Published:** 2019-02-05

**Authors:** Gian Maria Ragucci, Basel Elnayef, Fernando Suárez-López del Amo, Hom-Lay Wang, Federico Hernández-Alfaro, Jordi Gargallo-Albiol

**Affiliations:** 1 Department of Oral and Maxillofacial Surgery, International University of Catalonia, C/Josep Trueta Sn, Sant Cugat del Vallés, C.P 08195 Barcelona, Spain; 2 Department of Periodontics, University of Oklahoma Health Sciences Center – College of Dentistry, Oklahoma City, OK USA; 3 Department of Periodontics and Oral Medicine, University of Michigan School of Dentistry, Ann Arbor, MI USA

**Keywords:** Maxillary sinus, Dental implant, Maxillary sinusitis, Sinusitis, Bone regeneration, Bone grafting

## Abstract

**Background:**

After tooth loss, the posterior maxilla is usually characterized by limited bone height secondary to pneumatization of the maxillary sinus and/or collapse of the alveolar ridge that preclude in many instances the installation of dental implants. In order to compensate for the lack of bone height, several treatment options have been proposed. These treatment alternatives aimed at the installation of dental implants with or without the utilization of bone grafting materials avoiding the perforation of the Schneiderian membrane. Nevertheless, membrane perforations represent the most common complication among these procedures. Consequently, the present review aimed at the elucidation of the relevance of this phenomenon on implant survival and complications.

**Material and methods:**

Electronic and manual literature searches were performed by two independent reviewers in several databases, including MEDLINE, EMBASE, and Cochrane Oral Health Group Trials Register, for articles up to January 2018 reporting outcome of implant placement perforating the sinus floor without regenerative procedure (lateral sinus lift or transalveolar technique) and graft material. The intrusion of the implants can occur during drilling or implant placement, with and without punch out Schneiderian. Only studies with at least 6 months of follow-up were included in the qualitative assessment.

**Results:**

Eight studies provided information on the survival rate, with a global sample of 493 implants, being the weighted mean survival rate 95.6% (IC 95%), after 52.7 months of follow-up. The level of implant penetration (≤ 4 mm or > 4 mm) did not report statistically significant differences in survival rate (*p* = 0.403). Seven studies provided information on the rate of clinical complications, being the mean complication rate 3.4% (IC 95%). The most frequent clinical complication was epistaxis, without finding significant differences according to the level of penetration. Five studies provide information on the radiographic complication; the most common complication was thickening of the Schneiderian membrane. The weighted complication rate was 14.8% (IC 95%), and penetration level affects the rate of radiological complications, being these of 5.29% in implant penetrating ≤4 mm and 29.3% in implant penetrating > 4 mm, without reaching statistical significant difference (*p* = 0.301).

**Conclusion:**

The overall survival rate of the implants into the sinus cavity was 95.6%, without statistical differences according to the level of penetration. The clinical and radiological complications were 3.4% and 14.8% respectively. The most frequent clinical complication was the epistaxis, and the radiological complication was thickening of the Schneiderian membrane, without reaching statistical significant difference according to the level of implant penetration inside the sinus.

## Introduction

The edentulous posterior maxillary region often presents with unique challenging conditions in implant dentistry [1]. Limited bone height secondary to pneumatization of the maxillary sinus and the resorption of the alveolar ridge preclude in many instances the installation of dental implants. To compensate for the lack of bone height, several treatment options have been proposed.

The most conservative and minimally invasive technique is the placement of short implants with less technically demanding operation, lower expense, fewer surgical procedures, and fewer complications [2–6]. Another therapeutic alternative is to use the residual, taking advantage of the residual bone, present in the anatomic buttress, as the frontomaxillary, frontozygomatic, and pterygomaxillary buttress [7], using zygomatic implants or pterygoid implants, combined with anterior standard implants; both of them have reported good survival and success rate [8–10]. Another alternative is regenerative procedure of the maxillary sinus encompassed by two main approaches: the lateral windows approach and the transalveolar or crestal approach. The technique of sinus augmentation was first published in 1980 by Boyne and James [11] and subsequently by Tatum [12]. It is most often used when severe degree of resorption is present, which precludes the installation of short implants and/or the achievement of primary stability. The transalveolar or crestal approach was first described by Summers [13] in 1996. This approach is commonly used when the degree of resorption is lower, and it is possible in the installation of dental implants with primary stability. Both techniques have shown high survival rates [14] similar to those implants installed in the non-grafted posterior maxilla [15].

The maxillary sinus is a paired pyramid-shaped paranasal cavity lined with thin respiratory ciliated epithelium that serves in the transportation of fluid secretions toward the ostium. This lining of the maxillary sinus cavity is called the Schneiderian membrane [16]. The integrity of the membrane is of paramount importance for the performance of sinus augmentation procedures and the avoidance of potential complications [17, 18]. However, perforation of the membrane remains as the most commonly occurring complications approximately in 15.7% of the cases [19]. Moreover, this complication can occur inadvertently. However, some evidence suggested that these perforations seem not to have a detrimental effect on implant survival or the appearance of further complications. In fact, recent investigations have found greater vital bone when perforations occurred [20]. As such, the survival rate does not differ between implants placed in perforated and non-perforated sinuses not statistically significantly different [21–23].

Intrusion of dental implants into the maxillary sinus perforating through the Schneiderian membrane is considered a cause of undesirable complications [24, 25]. However, this phenomenon has never been properly evaluated and systematically studied. For this reason, the aim of this systematic review was to assess the implant survival and complication rates of implants intruding into the sinus cavity.

## Materials and methods

This systematic review and subsequent meta-analysis follow the guidelines of the PRISMA statement.

### Focus question

**Fig. 1:**
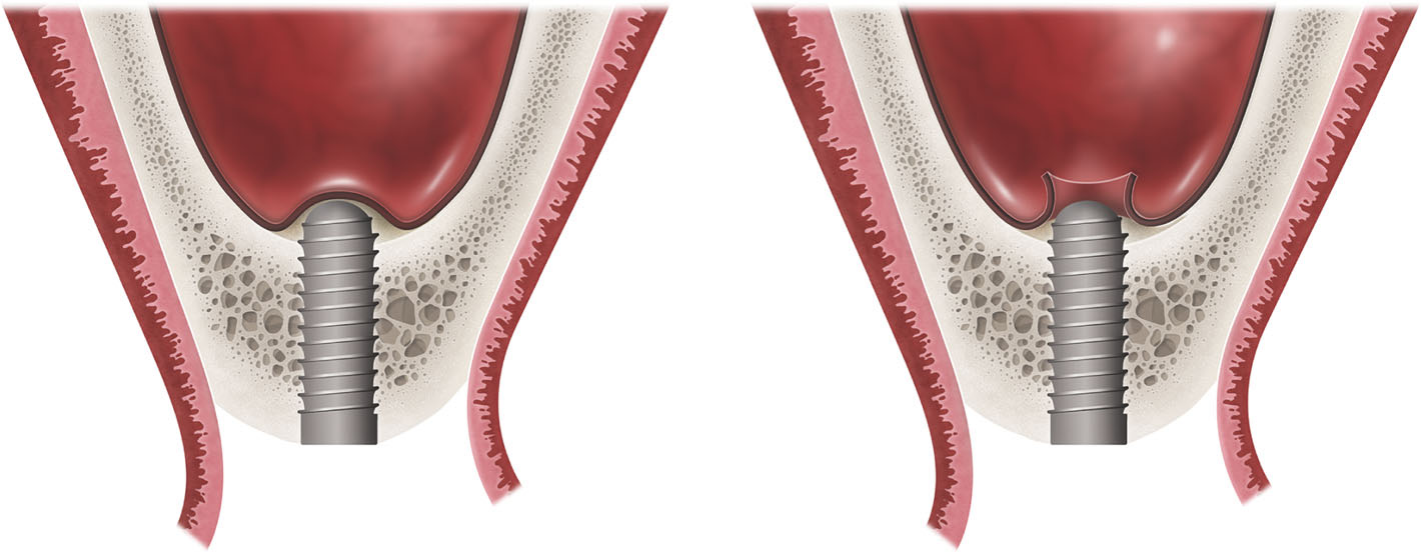
Graphic representation of implants intruding sinus perforating or not the Schneiderian membrane

The following focus question was developed: Is the intrusion of dental implants into the sinus cavity during implant drilling or implant placement, without regenerative procedure (lateral sinus lift or transalveolar technique) and graft material, has an effect on implant survival or increase clinical and radiographic complications? (Fig. 1) (Table 1).

**Table 1:** Clinical and radiographic complications reported in the studies

Clinical complications	Radiographic complications
Sinusitis	Thickening of Schneiderian membrane
Nasal bleeding, nasal obstruction, nasal secretion	Bone reaction to the implants
Headache and pain or tenderness in the region of the sinus	Sinus pathology
Decreased sense of smell	

Implant survival was defined as no pain on function, no mobility, 2–4 mm radiographic bone loss, and no exudates history.

Implant success was defined as no pain or tenderness upon function, no mobility, +2 mm radiographic bone loss from initial surgery, and no exudates history.

### Selection study

An electronic literature search was conducted by two independent reviewers (GMR and BE) based on the inclusion criteria in several databases, including MEDLINE, EMBASE, the Cochrane Central Register of Controlled Trials, and the Cochrane Oral Health Group Trials Register databases, covering articles written in English up until January 2018. Because no randomized controlled trials were found in the screening process, the included studies were analyzed with Newcastle-Ottawa scale (NOS). Both reviewers compared decisions, and their eligibility for this review was confirmed after discussion. Full articles were obtained for all the investigations deemed eligible for inclusion in this paper and further evaluated by both reviewers. If needed, a third reviewer was consulted to ensure consensus.

### Screening process

Literature search was conducted in several databases including MEDLINE (PubMed) and EMBASE from 1980 to 2018. All article titles and abstracts were screened in order to eliminate non-qualifying studies. Next, full-text evaluation of each article was performed in order to confirm the eligibility based on the inclusion and exclusion criteria. Combinations of controlled terms (MeSH and EMTREE) and keywords were used whenever possible, with “[mh]” representing the MeSH terms. In addition, other terms not indexed as MeSH and filters were applied. The key terms used were the following: (((((((((((((“maxillary sinus”) OR “schneiderian membrane” [MeSH Terms]) OR “schneiderian membranes” [MeSH Terms]) OR “dental implant” [MeSH Terms]) AND “perfor*”) OR “penetrat*”) OR “intruding*”) OR “sinus perforat*” OR “membrane perforation*”) OR “schneiderian membrane peforation*”). References of full-text-evaluated investigations were also screened. In addition, a manual search in periodontics/implantology-related journals, including “Clinical Oral Implant Research,” “Journal of Dental Research,” “Journal of Clinical Periodontology,” “Journal of Periodontology,” “Clinical Implant Dentistry and Related Research,” and “The International Journal of Periodontics & Restorative Dentistry,” from 1980 up to 2018, was also performed to ensure a thorough screening process.

#### Eligibility criteria

Articles were included in this systematic review if they met the following inclusion criteria: human prospective or retrospective studies, reporting outcomes of implant placed perforating the sinus floor with implant burs, and without regenerative procedure (lateral sinus lift or transalveolar technique) and graft material. The intrusion into the sinus cavity can occur during drilling or implant placement, with and without punch out Schneiderian membrane. Only studies with at least 6 months of follow-up were included in the qualitative assessment.

The following articles were excluded: case report and case series; animal studies; systematic reviews; in vitro studies; human studies using grafting materials, lateral access sinus lift, and transalveolar technique; and follow-up less than 6 months.

#### Risk of bias

Two reviewers (GMR and BE) designed and assessed the proposal for the present project to make sure that the STROBE statement and PRISMA guideline were followed. STROBE stands for an international, collaborative initiative of epidemiologists, methodologists, statisticians, researchers, and journal editors involved in the conduction and dissemination of observational studies, and consists of a 22-item checklist to be fulfilled in a systematic review.

#### Qualitative assessment

The quality of the selected randomized controlled trials (RCTs) was established from the randomized clinical trial checklist of the Cochrane Center and CONSORT (Consolidated Standards of Reporting Trials) statement, which provided guidelines for the following parameters: (1) sequence generation, (2) allocation concealment method, (3) masking of the examiner, (4) address of incomplete outcome data, and (5) free of selective outcome reporting. The Newcastle-Ottawa scale (NOS) was used to assess the risk of bias of non-randomized studies. This was performed by two investigators (GMR and BE). Cohen ´s kappa coefficient was used to assess inter-rater agreement.

#### Statistical analysis

The R 3.0.2 software package was used to perform the meta-analysis. The primary variable was implant survival rate. The secondary variable was the relationship between the degree of penetration and clinical and radiological complication. The analysis was performed using the methodology described below. The pooled weighted mean (WM) and the 95% confidence interval (CI) of each variable were estimated using a computer program (Comprehensive Meta-analysis version 2, Biostat). Random effects meta-analyses of the selected studies were applied to account for potential bias arising from methodology.

#### Study of heterogeneity

Heterogeneity was assessed based on calculation of the *I*^2^ statistic (percentage variability of estimated effect that can be attributed to the heterogeneity of the effects) and the null statistic test. Galbraith graphs displayed the degree of heterogeneity. In studies where great heterogeneity was detected, a sensitivity analysis was performed to determine its source. Funnel plots and the Egger test were used to assess risk of bias of the accepted statistical significance level of 5% (*p* = 0.05).

## Results

### Study screening

**Fig. 2:**
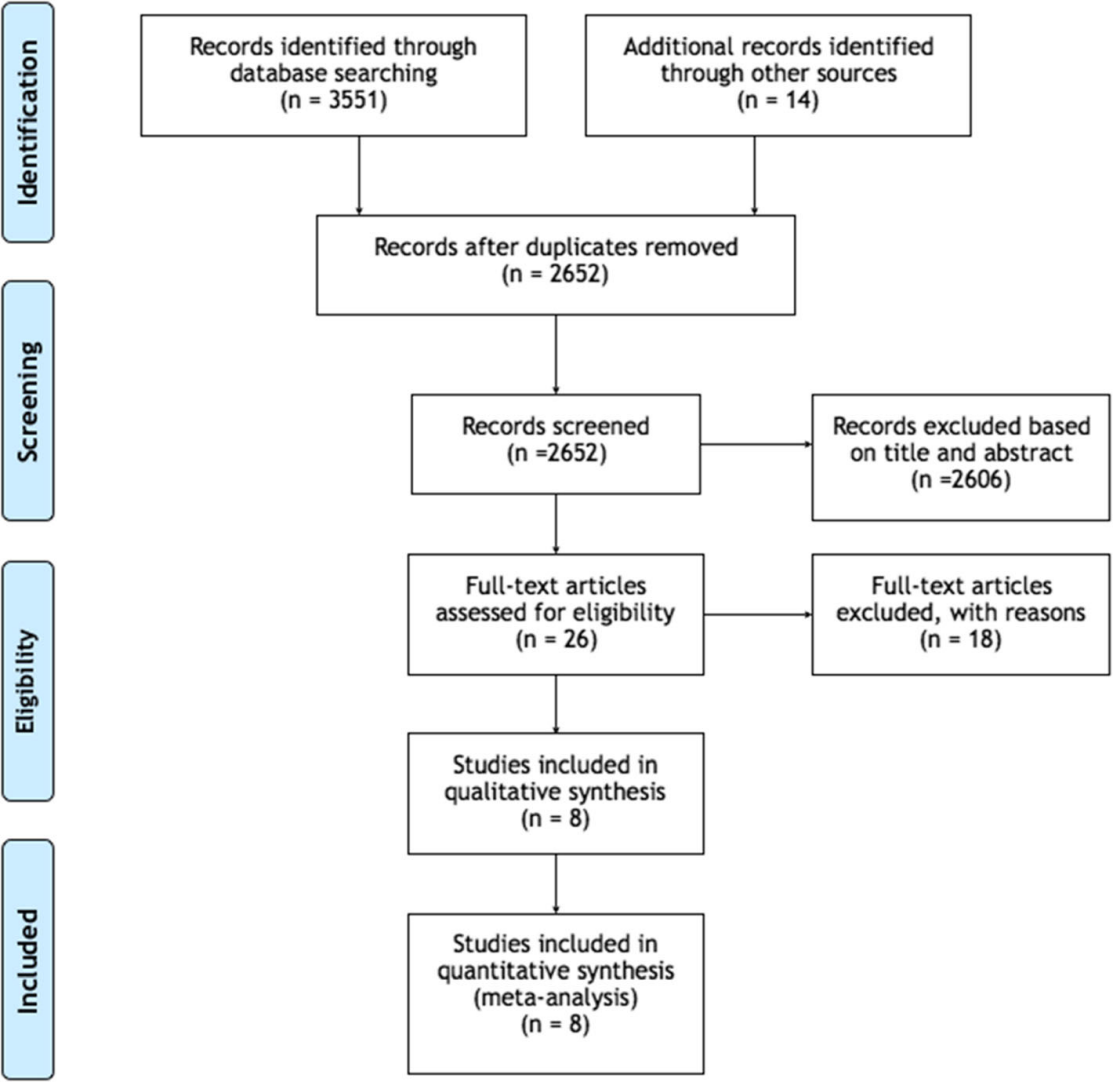
PRISMA flowchart of the screening process

An initial screening yielded a total of 3551 publications of which 26 potentially relevant articles were selected after an evaluation of their titles and abstracts. Full text of these articles was obtained and evaluated thoroughly. Of these, eight articles [26–33] (Table 2) fulfilled the inclusion criteria and subsequently were included in the qualitative analysis (Fig. 2). Reasons for exclusion are displayed in supplementary (Table 3).

**Table 2:** Characteristics of the included investigations

Author (year)	Study design	Follow-up (months)	*N* of patients	*N* of implants	Smokers	Length and diameter (mm)	Implant system
Shihab 2017 [33]	Retrospective	60	35	70	NA	5–12 × 3.0–5.7	IDI FMD Nucleoss
Ghanem 2014 [32]	Retrospective	72	10	10	NA	NA	NA
Nooh 2013 [31]	Prospective	12	56	63	0	4 × 8 4.3 × 10 5 × 8 5 × 10	Nobel Biocare
Kim 2013 [30]	Retrospective	17.9	39	87	NA	8-9-10-11,5 × 4–5	NA
Abi Najm 2013 [29]	Retrospective	118	70	83	7	NA	Strauman
Tabrizi 2012 [28]	Retrospective	12	13	18	NA	NA	Astra tech Zimmer DIO
Jung 2007 [27]	Retrospective	10	9	23	NA	NA	Astra tech Osstem implant
Branemark 1984 [26]	Retrospective	120	101	139	NA	NA	Branemark system
1–2 phase	Graft material	CBH (mm)	Penetration (mm)	Evaluation	ncm	Membrane perforation (%)	Clinic complications (Pat level)
2	No	≤ 4	> 4	rx(opg)-clinic	> 30	100	2.1% epistaxis
2	No	NA	≤ 4	rx(opg-MSCT)-clinic	NA	100	0%
2	No	5–8	< 4	rx(opg)-clinic	> 25	100	12.5% epistaxis 1.78% sinusitis
2	No	4.2–9.3	1–5	rx (opg)-clinic	25	100	7.7% epistaxis
1	No	5–8	< 4	rx (periapical-opg)-clinic	NA	100	0%
NA	No	NA	< 4	rx (periapical-CT)-clinic	NA	NA	0%
2	No	NA	> 4	rx(CT)-clinic.questionare	NA	NA	0%
2	No	NA	NA	rx-clinic	NA	NA	NA
Rx complications	Type of prosthesis	Implant failure before loading	Implant failure after loading	Total implant failure	Survival rate (%)	Bone loss (mm)	
0%	NA	1	1	2	97%	0	
0%	49 SC	0	0	0	100%	NA	
NA	49 Unilateral SC 7 Bilateral SC	1	0	1	98%	NA	
NA	31 SC 56 splinted for FA	0	0	0	100%	+ 0.05	
0%	NA	0	0	0	100%	NA	
16% (patients) thickening membrane	NA	0	0	0	100%	0	
60% (implants) thickening membrane	NA	0	0	0	100%%	NA	
NA	NA	NA	NA	30%	70%	NA	

*CHB* crestal bone height, *mm* millimeters, *SC* single crown, *FA* full arch restoration, *NA* not available, *rx* radiography, *opg* orthopantomography, *CT* computerized tomography, *MSCT* multi-slice computerized tomography

**Table 3:** Articles excluded and reasons for exclusion

Reason for exclusion	Investigations
Study design (case series or case report)	Kim et al. (2017), Hatano et al. (2007)
Different grafting technique (lateral sinus lift or transalveolar technique)	Jensen et al. (1994), Winter et al. (2002), Toffler et al. (2004), Chappuis et al. (2009), Soltan et al. (2011), Xiao et al. (2011), Cricchio et al. (2011), Scala et al. (2012), Bruschi et al. (2013), Kadkhodazadeh et al. (2013), Pjetursson et al. (2014), Monje et al. (2016), Falah et al. (2016), Markovic et al. (2017)
Type of implant (Zygoma implant)	Pjetursson et al. (2004)

The NOS was used to appraise the quality of included studies for a proper understanding of nonrandomized studies. Because no nonrandomized controlled trials were found in the screening process, the 17 included studies were analyzed with NOS. The level of agreement between the reviewers regarding study inclusion calculated using Cohen kappa statistic interagreement rate of 0.8 was reached. A mean NOS score of 5.1 ± 1.4 was obtained after discussing the disagreements between the examinees (GMR and BE) and third consultant (FL-A).

### Implant survival

**Fig. 3:**
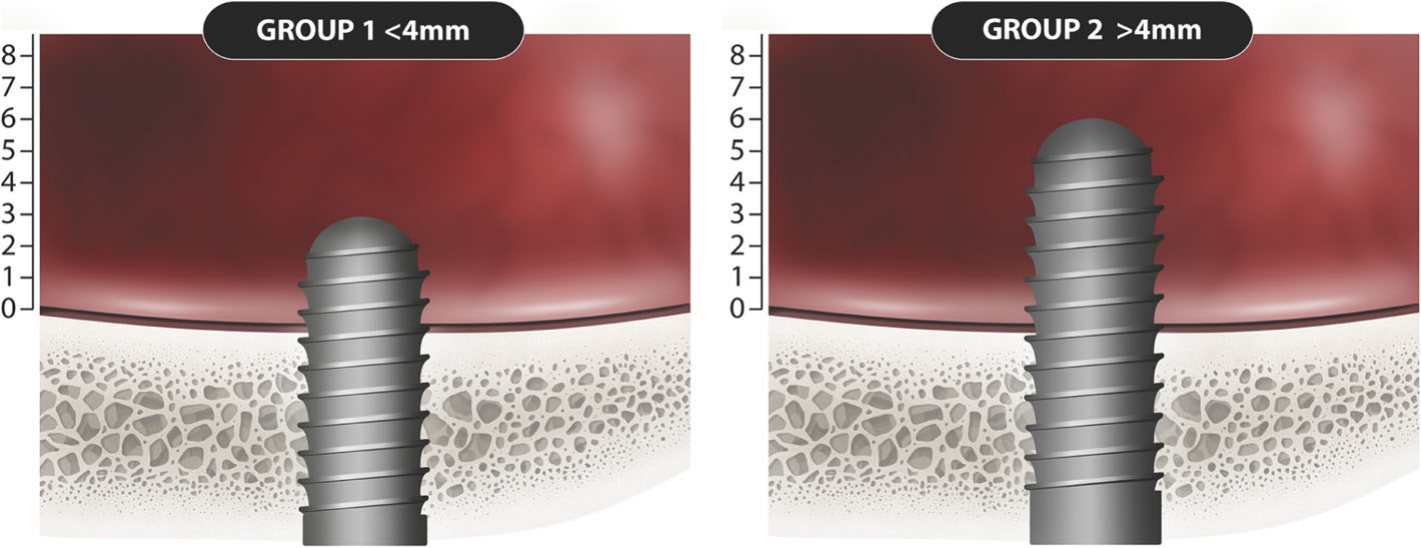
Graphic representation of group 1 ≤ 4 mm penetration and group 2 > 4 mm penetrations

**Fig. 4:**
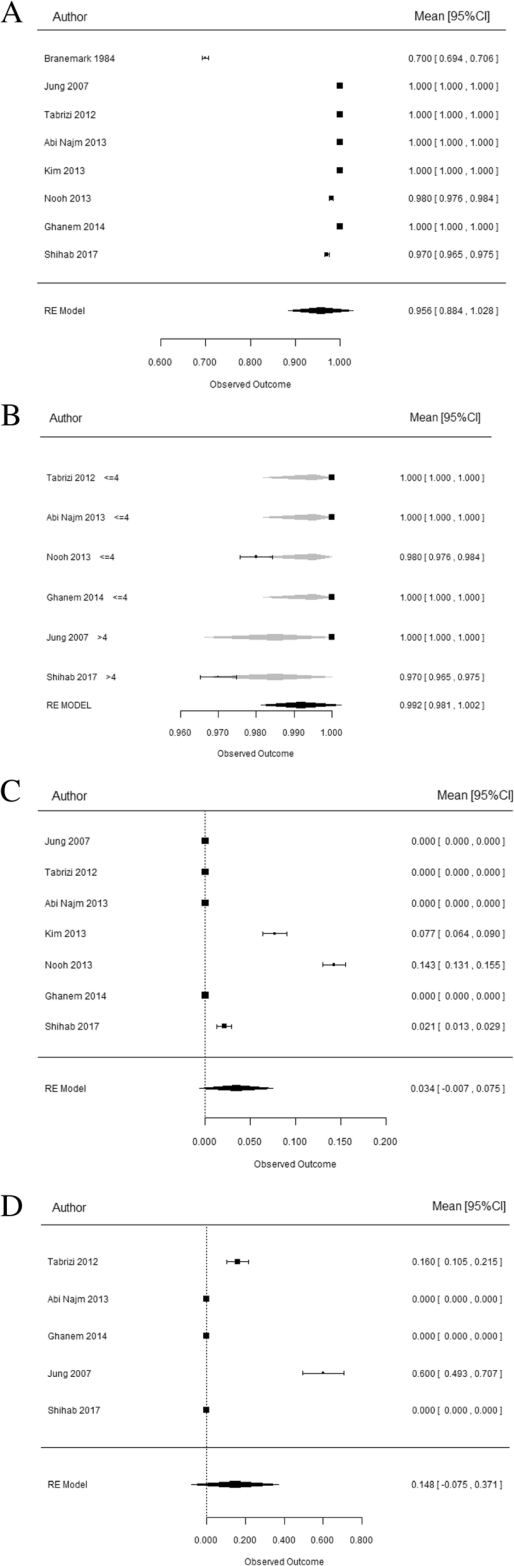
Statistical analysis for different variables. **a** Weighted mean survival rate. **b** Implant survival rate according to degree of penetration. **c** Analysis of clinical complications. **d** Analysis of radiographic complications

Eight studies [26–31, 33] (Table 2) provide information on the survival rate, which consisted of a global sample of 493 implants, of which a subtotal of 340 reported the degree of penetration (Fig. 3), with a mean follow-up of 52.7 months. The implant survival between the different authors range from 70 to 100%, being the weighted mean survival rate 95.6% with an IC 95% [88.4100] (Fig. 4a). A re-estimation is proposed excluding Brånemark et al. [26] from the meta-analysis, due to the year of publication which is before 2000, implant surface, and the surgical technique that can lead to these low survival rates of 70%. The seven articles [27–33] of the last 10 years remain in the calculations, being the weighted survival rate of 99.3% with an IC 95% [98.4100]. With regard to the influence of the penetration level, it was categorized into two levels with a cutoff point of 4 mm (Fig. 4b). It was analyzed if there were any differences in survival according to this aspect. For this, six studies contribute with 267 implants [27–33]. The estimated survival rates were 99.5% CI [98.2100] in implant penetrating ≤ 4 mm and 98.5% CI [96.6100] in implant penetrating > 4 mm (Fig. 3). There were no statistical significant differences in survival according to the degree of penetration (*p* = 0.403) (Fig. 4b).

### Analysis of clinical complications

Seven studies [27–33] provide information on clinical complications with a global sample of 232 patients. Clinical complications among the different authors range from 0 to 14.3%, being the weighted mean complication rate 3.4% with an IC 95% [0 7.5] (Fig. 4c) Clinical complications analyzed in the studies were sinusitis, nasal bleeding, nasal obstruction, nasal secretion, mucopurulent drainage, headache and pain or tenderness in the region of the sinus, facial pain-pressure-fullness, and decreased sense of smell. The most common clinical complication was epistaxis, reported in three studies [30, 31, 33], followed by sinusitis, reported in only one study [31]. Other complications did not appear. With regard to the influence of penetration level, 193 patients can be included in the analysis. The estimated complication rate was 3.54% CI [0 9.62] in implant penetrating ≤ 4 mm and 1.05% IC [0 9.63] in implant penetrating > 4 mm. The differences were not statistically significant in the rate of clinical complications according to the degree of penetration (*p* = 0.642).

### Analysis of radiographic complications

Five studies [27–29, 32, 33] provide information on the radiographic complication rate with a global sample of 137 patients. Complications between the different authors range from 0 to 60%, being the weighted complication rate 14.8% with an IC 95% [0 37.1](Fig. 4d). Radiographic complications analyzed in the articles were thickening of the Schneiderian membrane, bone reaction to the implant, and any sinus pathology. The most common radiographic complication was thickening of the Shneiderian membrane, reported in two studies [27, 28] in 16% of patients in Tabrizi et al. [28] study and 60% of the implants in Jung et al. [27] study. Other complications did not appear.

With regard to the influence of the penetration level, the estimated complication rates are 5.29% CI [0 33.8] in implant penetrating ≤ 4 mm and 29.3% CI [0 64.6] in implant penetrating > 4 mm. There were no statistical significant differences in the radiographic complication rate according to the degree of penetration (*p* = 0.301).

## Discussion

Pneumatization of the maxillary sinus and resorption of the residual alveolar ridge following tooth extraction can compromise the dental implant placement. Similarly, extension of the dental implants inside the maxillary sinus cavity is not rare. Some studies have observed some differences in relation to the depth of the implant extension inside the sinus cavity. When the implants penetrate inside of the sinus cavity less than 2 mm, spontaneous covering of the implants with the sinus mucosa seems to occur [34, 35]. Also, new bone formation above dental implants has been described, especially if the implants exposed to the maxillary sinus do not tear the Schnederian membrane [34–38]. Nevertheless, when the implant extension inside the maxillary sinus is greater (more than 2 mm), the maxillary membrane do not repair spontaneously and debris accumulate on the exposed surfaces of the implants that were not covered by bone inside the antral cavity [34], which could lead to sinusitis. However, the long-term consequences of debris accumulation over the implants extended inside the maxillary sinus and perforating the Schneiderian membrane were not systematically evaluated before.

In relation to the long-term consequence of these different levels of implant protrusion, it was observed in this review that there were no statistically significant differences in implant survival, between implant penetrating ≤ 4 mm or > 4 mm, with a survival rate of 99.5% and 98.5% respectively.

Survival rate of the present review is in accordance with survival rates reported in a systematic review by Corbella et al. [39] in which the analyzed survival rates of different techniques for the treatment of atrophic posterior maxilla were as follows: short implants showed a survival rate from 86.5 to 98.2%, osteotome technique showed a survival rate from 95.4 to 100%, and sinuses augmentation through lateral technique showed an implant survival rate from 75.57 to 100%.

The secondary outcome of this review was the analysis of the clinical and radiological complications related to the penetration of implants in the maxillary sinus. Clinical complication among the different authors ranges from 0 to 14.3%, with a weighted mean complication rate of 3.4%, without finding statistical difference according to the level of implant penetration. The most common clinical complication was epistaxis, which can be considered a minor complication and that did not lead to major complications. Radiographic complication has also been shown low, weighted complication rate of 14.8%, without finding statistical difference according to the level of implant penetration. The most common complication was thickening of sinus membrane without having relevance at the clinical level. This is in concordance with the mongrel-dog study of Jung et al. [35], who observed after 6 months follow-up that the mucosa in the maxillary sinus cavity showed no inflammatory signs when dental implants perforating inside the maxillary sinus, suggesting that the extending implants do not make the maxillary sinus vulnerable to complications and do not cause any effect to the sinus physiology and resulting with no local or systemic pathology at all.

Consequently, it seems that maxillary sinus changes in relation to protruded implants inside the sinus cavity and does not statically affect to implant survival rate neither to clinical nor radiographic complications.

Several limitations could be described for the present review. Firstly, there is a lack of a control group in the included studies, to compare outcomes and complications, with implant placed in native bone, or with regenerative techniques. Second limitation was related to the types of included studies being seven retrospectives [26–33] and one prospective study [31]. Third, the lack of a reliable method to evaluate the millimeters of implants intruded inside the maxillary sinus and to assess sinus membrane perforation or not. Fourth, the analysis of the radiographic complications has been analyzed only in three studies with computerized tomography and in two studies by orthopantomographies and periapical radiographs, taking into account the difficulties of analyzing radiological complications in periapicals and orthopantomographies.

Future research should focus on performing randomized studies comparing implant intrusion in the maxillary sinus versus regenerative techniques, focusing on the rate of complications and patient outcomes. Further, it would be interesting to study the possibility of placing short implants and thus avoid access to the maxillary sinus.

## Conclusions

The current review showed that the exposure of dental implants in the sinus cavity without the augmentation procedure or graft materials shows a high survival rate of 95.6%, without statistically significant differences according to the level of penetration (lower or higher to 4 mm). Changes in maxillary sinuses in relation to protruding implants within the sinus cavity do not statically affect clinical or radiographic complications of 3.4% and 14.8% respectively. Although caution is necessary, it is not advised to carry out this technique intentionally, since the supporting literature is based only on retrospective studies. Further research is needed, with prospective and randomized studies that directly compare different techniques in equal local and systemic conditions to explore the complications and outcomes of the patient center.
